# Repeated closed-head mild traumatic brain injury-induced inflammation is associated with nociceptive sensitization

**DOI:** 10.1186/s12974-023-02871-1

**Published:** 2023-08-27

**Authors:** Tyler Nguyen, Natalie Nguyen, Ashlyn G. Cochran, Jared A. Smith, Mohammed Al-Juboori, Andrew Brumett, Saahil Saxena, Sarah Talley, Edward M. Campbell, Alexander G. Obukhov, Fletcher A. White

**Affiliations:** 1 Department of Anesthesia, Indiana University School of Medicine, Indianapolis, IN USA; 2 Stark Neurosciences Research Institute, Indiana University School of Medicine, Indianapolis, IN USA; 3 Medical Scientist Training Program, Indiana University School of Medicine, Indianapolis, IN USA; 4 Department of Microbiology and Immunology, Loyola University Chicago, Maywood, IL USA; 5 Stritch School of Medicine, Loyola University Chicago, Maywood, IL USA; 6 Department of Anatomy, Cellular Biology, and Physiology, Indiana University School of Medicine, Indianapolis, IN USA

**Keywords:** Caspase-1, mTBI, Inflammation, Nociceptive sensitization

## Abstract

**Background:**

Individuals who have experienced mild traumatic brain injuries (mTBIs) suffer from several comorbidities, including chronic pain. Despite extensive studies investigating the underlying mechanisms of mTBI-associated chronic pain, the role of inflammation in long-term pain after mTBIs is not fully elucidated. Given the shifting dynamics of inflammation, it is important to understand the spatial-longitudinal changes in inflammatory processes following mTBIs and their effects on TBI-related pain.

**Methods:**

We utilized a recently developed transgenic caspase-1 luciferase reporter mouse model to monitor caspase-1 activation through a thinned skull window in the in vivo setting following three closed-head mTBI events. Organotypic coronal brain slice cultures and acutely dissociated dorsal root ganglion (DRG) cells provided tissue-relevant context of inflammation signal. Mechanical allodynia was assessed by mechanical withdrawal threshold to von Frey and thermal hyperalgesia withdrawal latency to radiant heat. Mouse grimace scale (MGS) was used to detect spontaneous or non-evoked pain. In some experiments, mice were prophylactically treated with MCC950, a potent small molecule inhibitor of NLRP3 inflammasome assembly to inhibit injury-induced inflammatory signaling. Bioluminescence spatiotemporal dynamics were quantified in the head and hind paws, and caspase-1 activation was confirmed by immunoblot. Immunofluorescence staining was used to monitor the progression of astrogliosis and microglial activation in ex vivo brain tissue following repetitive closed-head mTBIs.

**Results:**

Mice with repetitive closed-head mTBIs exhibited significant increases of the bioluminescence signals within the brain and paws in vivo for at least one week after each injury. Consistently, immunoblotting and immunofluorescence experiments confirmed that mTBIs led to caspase-1 activation, astrogliosis, and microgliosis. Persistent changes in MGS and hind paw withdrawal thresholds, indicative of pain states, were observed post-injury in the same mTBI animals in vivo. We also observed enhanced inflammatory responses in ex vivo brain slice preparations and DRG for at least 3 days following mTBIs. In vivo treatment with MCC950 significantly reduced caspase-1 activation-associated bioluminescent signals in vivo and decreased stimulus-evoked and non-stimulus evoked nociception.

**Conclusions:**

Our findings suggest that the inflammatory states in the brain and peripheral nervous system following repeated mTBIs are coincidental with the development of nociceptive sensitization, and that these events can be significantly reduced by inhibition of NLRP3 inflammasome activation.

**Supplementary Information:**

The online version contains supplementary material available at 10.1186/s12974-023-02871-1.

## Background

Mild concussive events or closed-head mild traumatic brain injuries (mTBIs) are frequent in young and aged populations and often occur due to a fall. Recurrent falls leading to mTBIs are a major problem as the events can often lead to impaired memory, depression, anxiety, and chronic pain [1, 2]. Though some of these conditions may be likely associated with white matter tract injury or diffuse axonal injury, often there are no detectable overt signs of neuropathology [3, 4]. Due to the apparent lack of actual tissue damage causing activation of peripheral nociceptors or lesion of the somatosensory system causing the pain, accumulating evidence suggests that the chronic pain is maintained by central sensitization.

Central sensitization can occur due to various factors, one of which is persistent inflammation. Inflammation can trigger central sensitization through the release of proinflammatory chemicals which lead to increased excitability of neurons in the spinal cord and brain [5]. Although not all cases of central sensitization are driven by inflammation, and not all instances of inflammation lead to central sensitization [5], treatment approaches targeting inflammation may sometimes help in managing nociceptive sensitization [6–8]. Despite extensive research efforts in basic and clinical science to date, the pathophysiology of the nociceptive sensitization associated with mTBI remains poorly understood.

Mechanisms which may contribute to mTBI-associated inflammatory responses include the release of endogenous mediators acting as danger signals [damage-associated molecular patterns (DAMPs)], such as high mobility group box-1 [9, 10]. Some of the numerous DAMPs released within the CNS after head trauma serve to trigger the assembly of intracellular multiprotein complexes called inflammasomes present in several cell types [11]. Inflammasomes are cellular complexes involved in regulating the inflammatory response and may be central to the initiation and perhaps maintenance of mTBI-associated nociceptive pain states [12].

The inflammasome is composed of nucleotide-binding oligomerization domain (NOD)-like receptor protein 3 (NLRP3) [13]. Notably, the NLRP3 inflammasome plays a critical role in activation of caspase-1 proteolytic enzyme [14], which in turn, contributes to the release of proinflammatory cytokines known to influence chronic pain such as interleukin-1β (IL-1β) and IL-18 [15, 16]. Though NLRP3 inflammasomeswere thought to be exclusive to microglia in the central nervous system, recent studies demonstrate that NLRP3 inflammasome complex may also form in other brain cells, including oligodendrocytes, astrocytes, and neurons [12, 17–19].

A recently developed transgenic mouse model globally expressing a caspase-1 luciferase-based reporter (biosensor) allows the detection of ongoing NLRP3 inflammasome-initiated inflammatory responses following mTBI in in vivo and ex vivo settings [20, 21]. The usage of this transgenic mouse model facilitates a temporal monitoring of the kinetics and magnitude of inflammatory responses in the brain and periphery of both male and female mice subjected to repetitive injury across time [20, 21].

Considering that both single and repetitive mTBI events can contribute to the onset of chronic pain behavior, we assessed the injury-induced changes in caspase-1 bioactivation across time in both the peripheral and central nervous system. Additionally, we tested the effects of the potent NLRP3 inflammasome-specific inhibitor MCC950 on mTBI-induced caspase-1 activation and behavioral responses. These findings highlight the importance of the activated caspase-1 and NLRP3 signaling in mTBI inflamed tissue and demonstrate the efficacy of MCC950 in inhibiting nociceptive events.

## Methods

### Animals

**Fig. 1:**
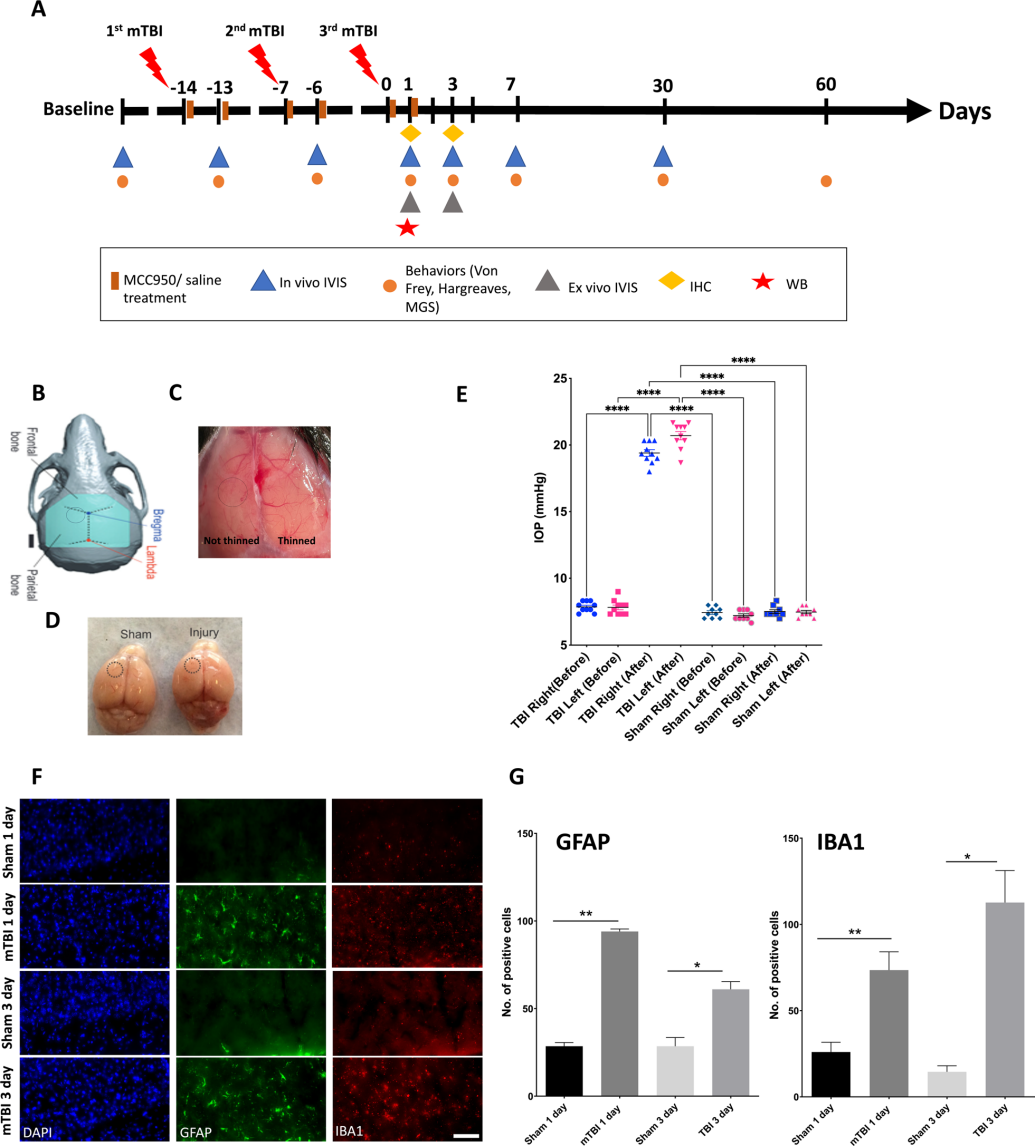
Cranial window and mTBI model preparation. **A** Experimental design timeline. Caspase-1 activation reporter mice were subjected to three closed-head mTBIs over a period of three weeks (each injury occurred one week apart). Corresponding IVIS imaging (in vivo and ex vivo), von Frey, Hargreaves, mouse grimace score (MGS) behavioral assessment, and protein analysis (immunohistochemistry and Western blotting) were performed as shown. **B**, **C** The mouse skull was thinned and strengthened by high-strength transparent surgical cyanoacrylate glue (blue area). Sample image shows the thinned-skull window position of the transparent cranial window (**C**). **C**, **D** The thinned cranial hemisphere becomes transparent (right, **C**) compared to the not-yet-thinned side (left, **C**). Unilateral closed-head mTBI was induced on an area (black circle **B**, **C**, and **D**) that was approximately 1 mm rostral to the anterior edge of the windows. No apparent tissue damage was observed (**C**). **E** Increases in intraocular pressure of the eye ipsilateral to the injury cortex immediately after impact. **F**, **G** Brain sections were obtained from four groups of C57BL mice, including sham control (1 day), sham control (3 days), 3 × mTBI (1 day), and 3 × mTBI (3 days). **E**. Sample confocal images of coronal cortical slices stained for DAPI, GFAP, and IBA1 for each group. **G** Quantification of GFAP (left) and IBA1 (right) positive cells showed increases in GFAP and IBA1 labeling at 1 day and 3 days after the third injury (*n* = 3 mice per group; *p < 0.05, **p < 0.01, One-way ANOVA, Tukey’s HSD). *Scale bar:* 50 μm

All experiments were approved by the Institutional Animal Care and Use Committee (IACUC) of the Indiana University School of Medicine and performed in accordance with the National Institutes of Health guidelines for the care and use of laboratory animals. WT C57BL/6 mice (5–7 weeks old, ~ 20–25 g, The Jackson Laboratory) and caspase-1 activation luciferase reporter transgenic mice (5–7 weeks old, C57BL/6 genetic background, bred in house [20]) were used in this study. The caspase-1 activation reporter mice globally express a biosensor containing a circularly permuted form of luciferase, in which the N- and C-terminal domains necessary for bioluminescence are physically separated by a flexible hinge region, making luciferase inactive [22]. Active caspase-1 effectively cleaves this hinge region, thus promoting reassembly and the consequent activation of luciferase. Activated luciferase, in turn, cleaves free D-luciferin in the presence of cytosolic ATP leading to a photon release. The resulting bioluminescent signal is quantified as “photons per second” and displayed as an intensity map in the region of interest with multiple images captured over time. Caspase-1 activation luciferase reporter transgenic mice [20] were used for in vivo inflammasome activation imaging and ex vivo brain slice imaging experiments. Wild-type C57BL/6 mice were used for behavioral analysis, immunoblotting, and immunofluorescence assessments. All mice were maintained under pathogen-free conditions and randomly divided into sham, injury, and multiple treatment groups at different time points following mTBI (Fig. 1A). Each experimental group contained 6–25 mice. Some of the mice were also used for immunoblotting and immunofluorescence staining experiments. All mice, regardless of injury state or drug treatment condition were subjected to cranial window preparation described below.

### Thinned-skull cranial window preparation

To create a thinned-skull transparent window for in vivo imaging, mice were anesthetized with 125–250 mg/kg tribromoethanol (intraperitoneally) and fixed on a small animal stereotaxic system [22]. A heating pad set at 37 °C was used to maintain body temperature under anesthesia. Incisions were made on the scalp to remove a rectangular portion of skin (~ 7 × 10 mm) and expose the underlying skull (Fig. 1B). The periosteum was removed, and the exposed skull surface was rinsed with sterile saline and dried with a combination of cotton and compressed air. For skull thinning, a sterile IRF 0.6 mm drill bit in a Microtorque Foredom K.1070 drill (Foredom Inc., Bethel, CT, USA) was utilized at ~ 3000–4000 rpm. A rectangular area (~ 3 × 4 mm) of skull tissue with respect to the midline was thinned. To thin the bone, the microdrill was used in sweeping motions, with the drill bit being angled nearly parallel to the skull surface; no direct downward pressure was applied. This drilling motion was performed uniformly and continuously from anterior to posterior. The thinness of the skull was checked by placing sterile saline over the thinned area and viewed under a dissecting scope (Fig. 1C). As the transition from the harder thick bone layer to the spongy bone layer (i.e., the “diploe”), the drilling speed was reduced to ~ 1000–2000 rpm. Once the microvasculature became clearing visible, the drilling was stopped. At this time, a sterile dental microblade was used to scrape gently on the thinned skull surface under saline to achieve final thinning and even out the surface area. An ideal optimal thinned skull thickness was approximately 20–40 μm. Once the thinned skull window was ready, a thin layer of cyanoacrylate glue (C1000, Ted Pella Science, Inc. Redding, CA, USA) was applied using a 1.0-mm glass pipette to cover the thinned-skull area completely. This helped protect the thinned-skull area, slow down bone regrowth, and, most importantly, maintain transparency of the thinned-skull area for long-term imaging. Animals were then allowed to recover one week before baseline imaging, mTBI injury paradigm or associated drug treatments.

### mTBI procedure

mTBI was inflicted using a technique described recently [22–24]. Briefly, mice were anesthetized with 2–4% isoflurane, and their heads were stereotactically ‘fixed’ with flat tip bars, but still allow for lateral and angular movement, with heat-pad support below the abdomen. Closed-head mTBI was produced using a control cortical impact (CCI) device with a modified impactor tip by attaching a 3-mm-diameter round silicone tip of 1.5 mm thickness to it (Benchmark Stereotaxic Impactor, Leica Impactor One, Meyer Instrument, St. Louis, MO). After the baseline point of the device was set by lowering the tip to the skull surface and the stage position was set to zero, the impactor was retracted, and the impact depth was set. The approximate center of the impact site was 1 mm posterior to the bregma and 0.5 mm lateral from the midline on one cortical hemisphere. The skull was struck with the impactor tip at a speed of 4 m/s to a depth of 2 mm. Sham injury involved the entire procedure described above except for CCI and was performed once a week for 3 weeks. All animals survived the injury without skull fracture or hemorrhage.

### Intraocular pressure (IOP) measurements

Intraocular pressure (IOP) changes in the mouse eyes were used to both confirm the severity of skull impact and verify the absence of skull fracture [25]. An ICARE TONOLAB (Vantaa, Finland) tonometer was used to measure IOP, which is a surrogate of changes in brain pressure. The IOP measurements were performed immediately before and after the injury for up to 30 min (Fig. 1E, *n* = 10 mice in each group). Average IOPs of three separate readings per time point were calculated for each animal.

### MCC950 treatment paradigm

MCC950 (a selective NLRP3 inhibitor; CP-456,773, CRID3, Invivogen) was dissolved in sterile saline (5 mg/ml). The drug was injected intraperitoneally into each mouse 1 h and 24 h after each CCI or sham impact at a dosage of 10 mg/kg as previously reported elsewhere [26–29]. A total of 6 MCC950 injections were performed for each animal over the course of each study. Animals were divided into two groups (mixed males and females): vehicle control and MCC950 (*n* = 10–15 animals/group).

### In vivo inflammasome activity detection using bioluminescence imaging with an In Vivo Imaging Systems (IVIS) SpectrumCT device

Dynamic bioluminescence images (BLIs) were acquired using an IVIS SpectrumCT (Perkin-Elmer Inc. USA) imaging system that accommodates up to 5 mice per session. Prior to imaging, the skin over the animals’ abdomen regions was shaved to minimize light scatter. Anesthetic induction was achieved with 2–4% isoflurane, and animals were administered d-Luciferin (Promega, Madison, WI, USA) at 150 mg/kg via an intraperitoneal injection. Mice were immediately transferred to the heated stage (40 ± 1 °C) of the IVIS Imager, placed on their abdomen, and sequentially imaged at 2 min intervals for 40 min. At the completion of the sequence, anatomical reference photos were acquired, permitting the generation of a set of overlaid images. Animals were imaged at baseline, 24 h post-injury, 3 days, 1 week, and 1 month after the last injury (Fig. 1A). Mice were randomly divided into two groups (mixed males and females) sham controls and injury (*n* = 20–25 animals/group)**.** An identical imaging paradigm was used for the mTBI/saline-treated and mTBI/MCC950-treated groups.

### Isolation and IVIS imaging of primary dorsal root ganglion (DRG) cell cultures in vitro

DRGs of caspase-1 activation reporter mice were harvested from the entire spinal column 24 h after the third injury (or 24 h after last sham surgery). DRG were then cultured as previously described by Pittman and colleagues [30]. In short, mice were euthanized by CO_2_ asphyxiation. The isolated DRGs were incubated in F-12 media containing 0.01% collagenase for 2 h in a 3% CO_2_ at 37 °C, and mechanically dissociated. Suspensions of dissociated DRG cells were added to multi-well plates previously coated with poly-D-lysine and laminin (~ 30,000 cells/well in 12-well plates).

Cells were grown in F-12 medium supplemented with 10% heat-inactivated horse serum, 2 mM/L glutamine, 50 μg/mL penicillin and streptomycin, 50 μM/L 5-fluoro-2-deoxyuridine, 150 μM/L uridine, and 250 ng/mL NGF and were maintained at 37 °C in a water-jacketed 3% CO_2_ incubator. Cell culture medium was replaced every 2 days. Imaging was done 3 days after initiating the cultures. On imaging day, culture medium was replaced in each well with 2 mL of fresh complete culture medium, and 100 µL of d-luciferin (20 mg/mL) was added to each well one minute prior to the start of the imaging series. Images were taken in three series as described for ex vivo brain slice IVIS experiments. For MCC950 treatment, 100 μL of MCC950 (10 mg/mL) were added to the same chamber to a final concentration of 0.5 μg/mL 5 min prior to the third imaging series. All IVIS imaging experiments with cultured DRG neurons were done in a controlled 37 °C and 3% CO_2_ imaging chamber.

### Brain slice preparations

Caspase-1 activation reporter mice were anesthetized with 125–250 mg/kg tribromoethanol (intraperitoneally) and decapitated with scissors. After the scalp was removed, the skull was cut along the mid-sagittal line, and the two flaps of bone covering both brain hemispheres were removed. The brain was then removed and immediately placed in an ice-cold 4 °C oxygenated sucrose artificial cerebral spinal fluid (s-ACSF) solution (206 mM/L sucrose, 2 mM/L KCl, 1 mM/L MgCl_2_, 2 mM/L MgSO_4_, 1.25 mM/L NaH_2_PO_4_, 26 mM/L NaHCO_3_, 10 mM/L D-glucose, 1 mM/L CaCl_2_). After 1–2 min, the brain was glued onto a cutting stage such that the cortex faced the approaching blade. A piece of agarose gel was used as a cushion against the ventral part of the brain to prevent movement while cutting. Slices 350–400 μm thick were cut with a vibratome (Leica VT1200S; Leica, Nusslock, Germany), while the brain was submerged in a 4 °C s-ACSF. The slices were then incubated at 32 °C for 1 h in a gridded chamber filled with oxygenated incubating i-ACSF (124 mM/L NaCl, 3 mM/L KCl, 2 mM/L MgSO_4_, 1.25 mM/L NaH_2_PO_4_, 26 mM/L NaHCO_3_, 10 mM/L d-glucose, 1 mM/L CaCl_2_) before imaging sessions and never stored longer than for 4 h. Each experimental group contained 3–4 animals during the mTBI-MCC950 ex vivo IVIS imaging studies.

### Ex vivo brain slice IVIS SPECTRUM inflammasome bioluminescence imaging

The imaging paradigm for ex vivo brain slices is identical to the in vivo IVIS imaging described above. Brain slices were kept in oxygenated gridded chambers (one slice per slot in ~ 1 mL i-ACSF) at ~ 27–32 °C during the duration of imaging. For each imaging session, images were collected first after addition of only d-luciferin, and then lastly after addition of MCC950. Brain slices were imaged at a 2-min interval for a total of 8 min for each series. 100 µL of d-luciferin (20 mg/mL) was added to each well one minute prior to the start of the first imaging series to a final concentration of 2 µg/mL. Finally, 100 µL of MCC950 (10 mg/mL) was added to the same chamber up to a final concentration of 1 µg/mL 5 min prior to the second imaging series.

### Immunofluorescence analysis of astrocytes and microglia activation

To determine potential glial reactivity and neuroinflammation induced by mTBIs, we used an antibody against ionized calcium binding adaptor protein (IBA1) to label microglia and an antibody against glial fibrillary acidic protein (GFAP) to label astrocytes [31, 32]. C57BL/6J (*n* = 4/group) mice were divided into three groups: sham control, injury 1 day, and injury 3 days. Tissue preparations were performed as follows: the mice were deeply anesthetized and transcardially perfused with PBS buffer, followed by 4% paraformaldehyde (PFA) at room temperature. The brains were then dissected and placed in 30% sucrose for 48–72 h at 4 °C. The tissue was then frozen and sectioned with a cryostat at a thickness of ~ 45 µm. Immunofluorescence staining of free-floating sections was performed by incubating them overnight with a primary antibody. For astrogliosis and microglial activation staining, anti-GFAP (mouse; 1:800, Sigma Aldrich G3893) and anti-IBA1 (goat; 1:200, ABCAM ab5076) antibodies were followed by goat anti-mouse Cy5 (1:500, Jackson Immuno) and donkey anti-goat 488 nm (1:1000, Fisher Scientific) secondary antibodies, respectively. A DNA stain, 4’,6-diamidino-2-phenylindole (DAPI; 1:10,000, Fisher Scientific), was added to the solution for a final 5 min as a nuclear counterstain. Finally, fluorescence signals were imaged using a Neurolucida imaging system. The number of positive IBA1 or GFAP cells were counted at three random regions of the cortical area of each brain slice. Average of 3–5 brain slices per animal (total of 3 animal/group) were counted. The data were analyzed by two blinded experimenters.

### Protein immunoblotting of caspase-1

Animals were divided into three groups: sham, 3 × mTBIs, and 3 × mTBIs with MCC950 treatment. MCC950 treatment was performed in the same manner as described above at 1 h and 24 h after each injury. 24 h after the third injury, animals were euthanized, and the cortical tissues were collected and then rapidly frozen in liquid nitrogen. Immunoblotting was performed on the same day after tissue collection. Protein isolation and immunoblotting were performed as previously described [21, 33]. In brief, cortical brain tissue samples were sonicated in lysis buffer (RIPA buffer; Alpha Aesar, Catalog #J62524) supplemented with EDTA 0.5 M (Fisher Scientific, Catalog #BP24821), phenylmethanesulfonyl fluoride 0.1 M (Sigma, Catalog #93482), and Halt Protease and Phosphatase Inhibitor Cocktail (Thermo Fisher Scientific, Catalog #78441). The samples were then cleared by centrifugation (12,000 rpm, 10 min, 4 °C). Sample concentrations were determined using the Pierce BCA Protein Assay Kit (Fisher Scientific, Catalog #23227) and a FlexStation3 microplate reader (Molecular Devices LLC, San Jose, California). Protein samples were heat-denatured in Laemmli Buffer (BioRad, Catalog #1610747) supplemented with 2-mercaptomethanol (Sigma, Catalog #M3148) for 10 min at 95 °C and then separated by SDS-PAGE with 4–20% precast gels (BioRad, Catalog #4561094). The transfer was performed on an Immuno-Blot PVDF membrane (BioRad, Catalog #1620177). The membranes were blocked for 30 min with a TBS-intercept blocking buffer (Li-Cor, Catalog #927-60001) and then incubated with antibodies against caspase-1 (Mouse, 1:1000, Adipogen, Catalog #AG-20B-0042-C100) and β-Actin (Rabbit, 1:1000, Abcam, Catalog # ab8227) overnight at 4 °C on a rocker. After incubation, the membranes were washed 3 times with TBST (150 mM NaCl, 10 mM TRIS-base, 0.1% Tween 20, Fisher Scientific), followed by 3 washes with TBS. The membranes were then probed with 800 nm donkey anti-mouse (1:5000, Li-Cor Catalog # AB_2716622) and 680 nm donkey anti-rabbit (1:5000, Li-Cor Catalog # AB_2716687) for 2 h at room temperature on a rocker and then washed 3 times with TBST and 3 times with TBS. Finally, antibody complexes were detected on a LI-COR Odyssey CLx (LI-COR Biotechnology, Lincoln, Nebraska) using Image Studio, and the data were analyzed using ImageJ software. Blots were cropped to get final representative images. A total of 4 animals per group were used for this experiment.

### Von Frey behavioral assessment

Mechanical sensitivity was measured using calibrated Von Frey filaments measuring 100 μm in diameter and capable of exerting bending forces of 5, 10, 20, and 40 mN which were applied to the plantar surface of the animal hind paws in succession with the gradually increasing bending force until paw withdrawal was observed. Then, the corresponding stimulation intensity was recorded. Each stimulus lasted approximately 1 s and had a 10–15 s intermission between applications. Von Frey testing was performed at multiple time points: baseline (before injury), 24 h after each injury, and 3 days, 1 week, 1 month, and 2 months after the last injury. The test was performed on both the hindpaw ipsilateral to the CCI event and the contralateral hind paw. The test was conducted by a research analyst who was blinded to treatments and group assignment. An identical behavioral paradigm was used for all mTBI/saline control and mTBI/MCC950-treated groups (total 5–7 animals per group). Once the series of responses were obtained, the 50% threshold was determined and threshold reported in millinewtons/force (mN) according to previously published methods [34]. The force withdrawal threshold was calculated using this formula WT = 10^(x*F+*B*)^, where WT = withdrawal threshold, *F* = paw withdrawal threshold calculated via the Chaplan method, and *B* is the linear regression of log (bending force) = *x* * Filament force number + *B*. Force response thresholds were calculated per time point for each animal and plotted as average withdrawal thresholds. The data were analyzed by two blinded experimenters.

### Thermal analgesia

To evaluate the foot withdrawal to thermal stimulus, the Hargreaves plantar test apparatus (Ugo Basile, Varese, Italy) was used as described previously [35]. Mice were placed on a 2 mm-thick-glass floor and a mobile infrared heat generator with an aperture of 10 mm was aimed at mouse’s hind paw under the floor. Following heat source activation, the reaction time (the withdrawal latency of hindpaw) was recorded automatically. Shorter withdrawal time indicates higher thermal hyperalgesia. The temperature of the glass floor was between 22.1–23.5 °C. Measurement of the withdrawal latency of the paw began after the mice were habituated to the testing environment (IR setting = 70%). The measurements were repeated three times, at 5 min intervals, on each paw. The averages of the three measures were employed as data. The data were analyzed by two blinded experimenters.

### Mouse grimace scoring (MGS)

The MGS was performed as previously described [36]. In brief, mice were individually placed in cubicles (9L x 5W x 5H cm) with two walls of transparent Plexiglas and two opaque side walls (to encourage mice to face either forward or backward). An experimenter, who was blinded to the animal groups, assessed and scored facial behavioral changes through five different actions units (AU): orbital tightening (narrowing of orbital area, a tightly closed eyelid, or an eye squeeze), nose bulging (display a bulge on top of the nose), cheek bulge (cheek muscle is contracted and extended), ear position (ears pulled back from their original position or laid flat against the head), whisker change (whisker bended back towards the ear). Each action unit is given the score of 0, 1, or 2 (0—AU not present, 1—AU moderately visible, 2—AU severe). Calculated average from all AUs for each animal is presented as data. The data were analyzed by two blinded experimenters.

### Analysis of IVIS imaging data

For in vivo animals, ex vivo brain slices, and in vitro DRG cultures (to provide visualization, segmentation, and time series quantification from the 10-min scan), BLI and anatomical reference images were imported into LivingImage software (Perkin-Elmer Inc. USA). Pseudocolored parametric overlays of BLI time series with anatomical reference images were dynamically constructed for each imaging session. Using the image of the time series with the peak light emission for each individual animal, ROIs were designated for the region (as shown in their respective figures). The extracted time series were then analyzed to determine the total photon flux [p/s] of the time course kinetics. Using this information, each BLI time course was integrated to calculate the area under the total photon flux curve (AUC_TPF_), which represents the total light emission corresponding to the aerobic metabolism of d-luciferin.

### Statistics

Minimum group sizes were based on power analysis of results of previous experiments. Final plots were developed in Microsoft Excel and GraphPad Prism 6 (GraphPad Software, La Jolla, California), and values are reported as the mean ± S.E.M. All statistical analyses were performed with GraphPad Prism 6 and Jmp Analysis 11 (SAS Institute Inc. 2013. Cary, NC). ANOVA analyses were used for comparing areas under the total photon flux curve for the same groups of animals across all time points, areas among the control, injury, and treatment groups in vivo (repeated measures), and among groups of ex vivo brain slices and in vitro cell culture (repeated measures), comparison of GFAP and IBA1 fluorescence-positive cell signals among groups, immunoblotting of caspase-1 among groups (one-way), Von Frey pain thresholds (repeated-measure ANOVA), thermal analgesia thresholds (repeated-measure ANOVA), and grimace scoring (repeated-measure ANOVA). For comparisons that yielded statistical significance, Tukey’s HSD post hoc analyses were applied for further comparisons between specific groups. Statistical analyses were performed in an observer-blinding fashion.

## Results

### Repetitive mild traumatic brain injuries increased inflammatory response in multiple regions of the body

**Fig. 2:**
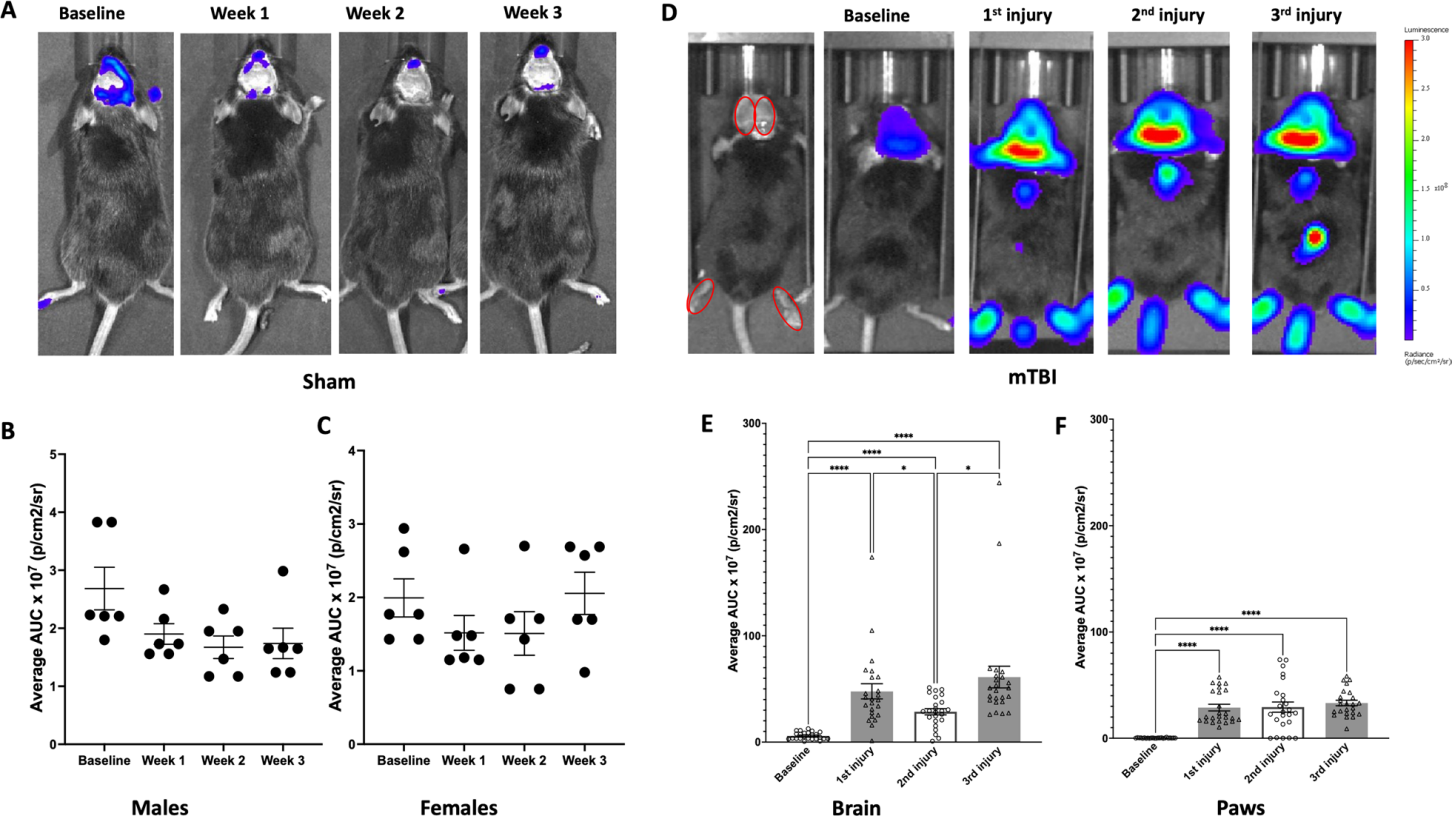
Concussive mild TBI increased caspase-1 activation biosensor bioluminescence intensity in the brain and hindpaw area of mTBI mice in vivo. **A–C** Thinned-skull cranial window enables long-term and consistent IVIS caspase-1 activation imaging. **A** Sample images show windowed caspase-1 activation biosensor mouse after window preparation (baseline) followed for three weeks post installation. **B**, **C** No change in caspase-1-mediated inflammatory signals in both male and female windowed animals. **D–F** Repetitive mTBIs promote caspase-1 activation bioluminescence IVIS signal. **D** Sample images show bioluminescence signals in caspase-1 activation reporter mice at 1 day after each injury. Injuries were separated by one week. The analyzed ROIs (red circles) were quantified across all time points as associated area under the curve (AUC) measurements. **E**, **F** Significantly elevated brain and paw caspase-1 signals in both injured mice were detected 1 day after each injury (*n* = 20–24 mice per group; **p < 0.05, ***p < 0.001, ****p < 0.0001, repeated-ANOVA, Tukey’s HSD)

Detection of inflammation states associated with diseases or injury has historically required tissue removal, preventing the real-time dynamic measurement of inflammatory or neuroinflammatory events in animals over time. To better understand the spatiotemporal kinetics following head trauma, we used a transgenic caspase-1 activation luciferase reporter mouse model together with a thinned-skull cranial window preparation to disseminate inflammatory dynamics within the same animals over time (Fig. 1A). To determine the effect of repetitive mTBIs on inflammation in vivo, we subjected mice to a thinned-skull cranial window procedure as described in our previous publications [37, 38] (Fig. 1B, C). These thinned-skull cranial windows were minimally invasive and could retain integrity following the physical impact generated during a concussive injury (Fig. 1D). To ensure thinned skull cranial windows did not affect caspase-1-mediated inflammation, we performed in vivo IVIS imaging of both male and female caspase-1 activation reporter mice following thinned-skulled window preparation. Animals were imaged 2 weeks after cranial window thinning followed by once-a-week imaging sessions for the following three weeks. We found low and stable IVIS inflammatory signal throughout the 5-week imaging period (Fig. 2A–C). This result indicates that the window preparation has minimal effects in the caspase-1 associated inflammatory response. For the remainder of the study, mice were subjected to a thinned-skull cranial window procedure followed 2 weeks later by a single mTBI event, once a week, for 3 weeks.

To confirm that the concussive event did not produce fracture to the skull or damage the cranial window preparation, we measured IOP changes in the mouse eyes before and post-injury. The closed-head injury produced a pronounced bilateral increase in intraocular pressure immediately after each CCI event (Fig. 1E). Thus, this CCI event could represent a clinical hallmark of concussion [39] but did not damage skull bone, did not affect vasculature in the underlying brain tissue, and did not cause any apparent tissue damage (Fig. 1D). We also observed significant increases in astrogliosis, as demonstrated by increases in the number of GFAP-positive cells in cortical tissues at day 1 and day 3 following the third injury event (Fig. 1F, G). Image analysis of IBA-1-immunopositive cells yielded evidence of microgliosis as defined by increased numbers of IBA1-immunopositive cells, which was also evident at day 1 and day 3 following the third injury event (Fig. 1F, G).

By utilizing the in vivo IVIS imaging approach, we first found that cranial window preparation has minimal effect on the bioluminescence inflammatory signal. We then observed a significant increase in bioluminescence intensity at 1 day after each injury compared to baseline (Fig. 2D–F). Remarkably, the  increase in caspase-1 activation-associated luciferase signals was not limited to the cephalic region (Fig. 2D, E), but was also observed in the hindpaws (Fig. 2D, F).

**Fig. 3:**
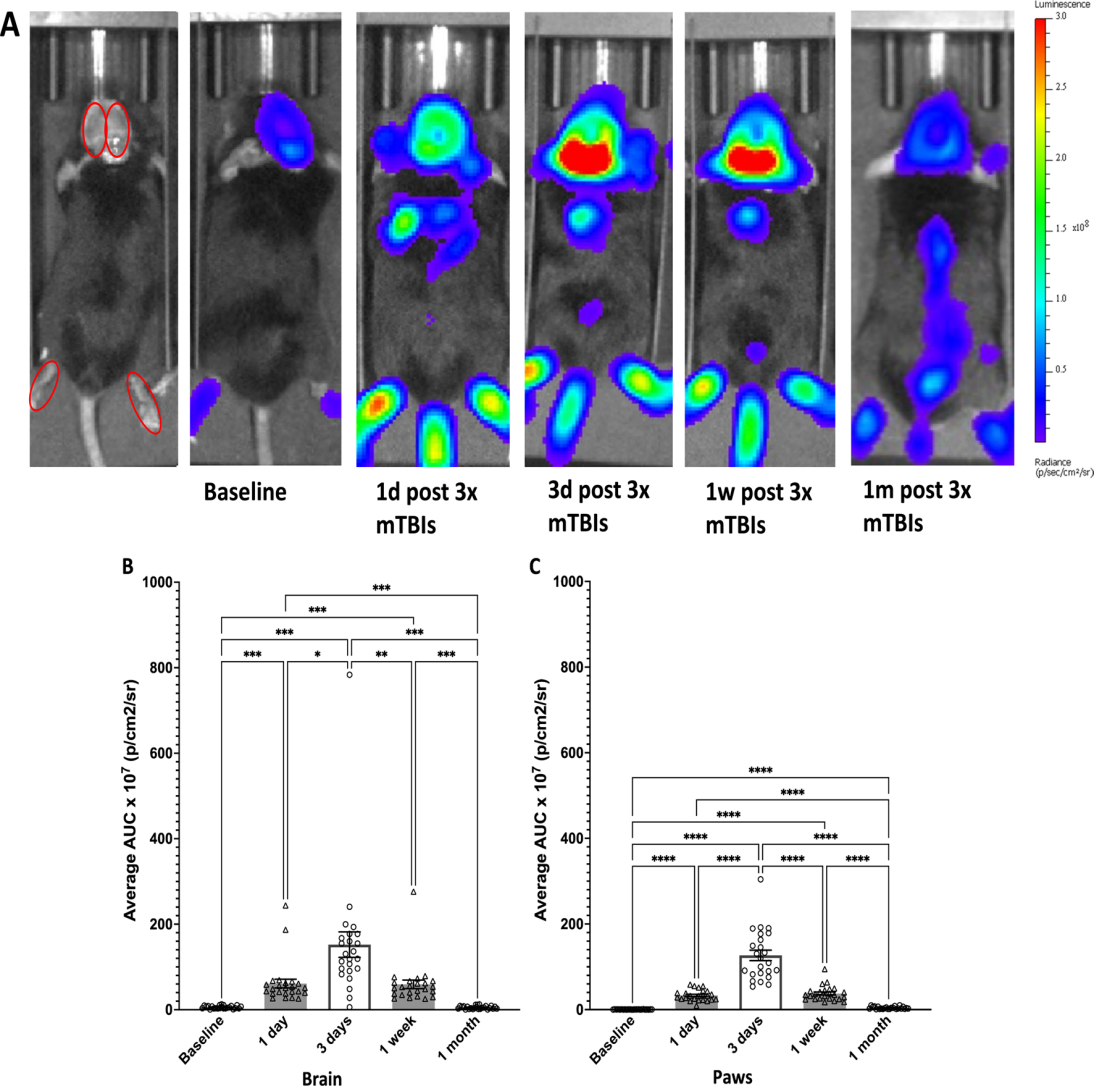
Repeated mTBI produced prolonged increase in caspase-1 activation reporter bioluminescence intensity in the brain and hindpaw area of mTBI mice up to at least one week after the last injury, in vivo. **A** Sample images show caspase-1 activation reporter mouse bioluminescence signals at 1 day, 3 days, 1 week, and 1 month after the last injury. The analyzed ROIs (red circles) were quantified across all time points. **B**, **C** Significantly elevated brain and paw caspase-1 activation-associated signals in injured mice were detected 1 day and sustained up to at least 1 week after the last injury (*n* = 20–24 mice per group; *p < 0.05, **p < 0.01, ***p < 0.001, ****p < 0.0001, repeated-ANOVA, Tukey’s HSD)

The long-term monitoring of caspase-1 activation-associated bioluminescence following the third injury revealed that signaling indicative of ongoing inflammation lasted for more than one week after injury in the brain region (Fig. 3A, B) and hindpaws (Fig. 3A, C). To confirm that the inflammatory signals originated from the brain tissue, we also performed IVIS imaging on freshly prepared brain slices from thinned skull mice subjected to the repetitive injury paradigm at 1 day and 3 days after the last injury. A robust increase in caspase-1-mediated bioluminescence signals was observed on both days (Additional file 1: Fig. S1A, B).

### Repetitive mild traumatic brain injury-induced mechanical allodynia

**Fig. 4:**
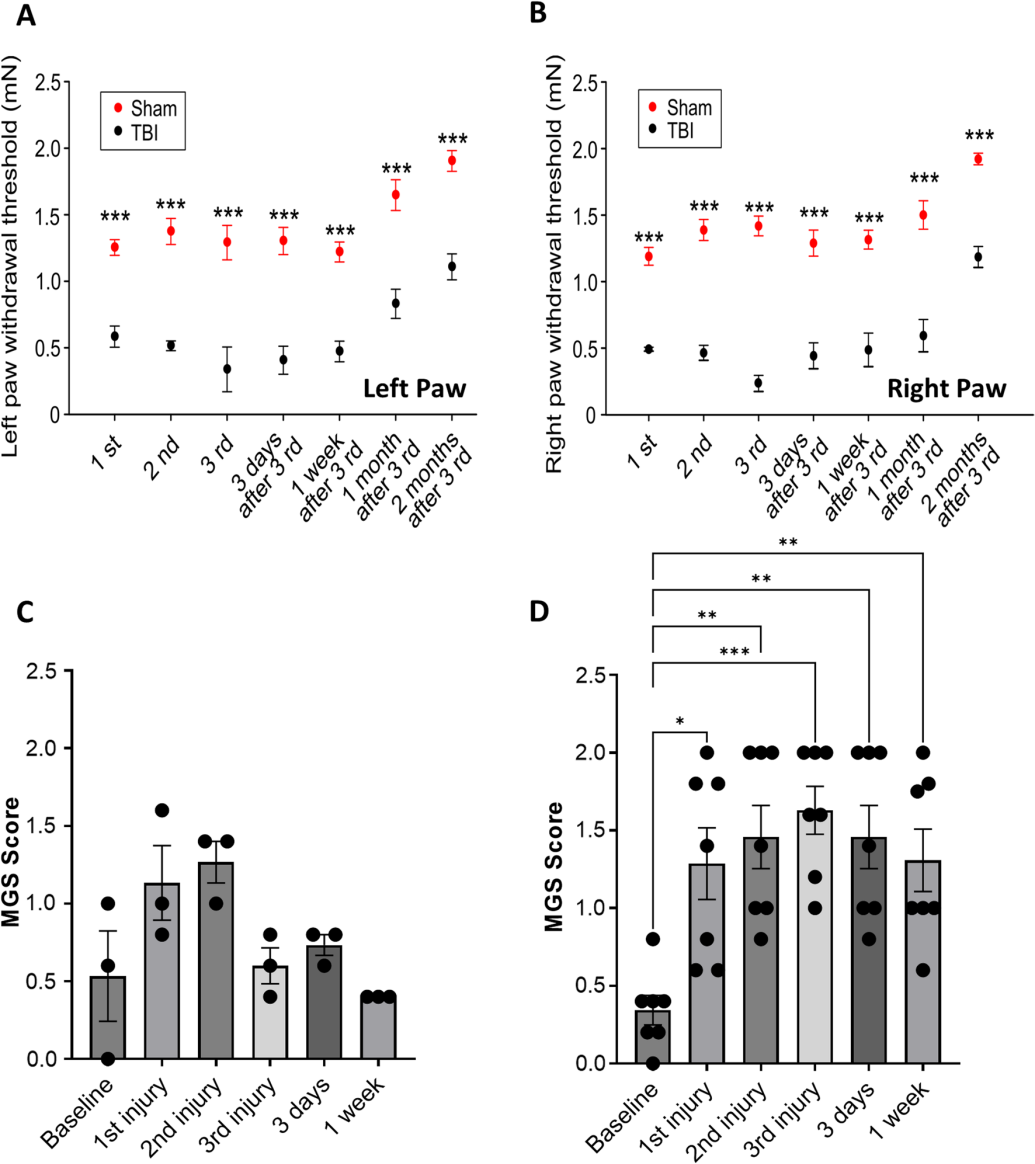
Repeated mTBIs produced robust increases in mechanical allodynia and grimace scores. Hypersensitive hindpaw withdrawal to tactile stimulus, ipsilateral to injured hemisphere (**A**, left hindpaw) and contralateral to injured hemisphere (**B**, right hindpaw), at 24 h after each injury and 3 days, 1 week, 1 month and 2 months after third injury. Statistically significant loss in response threshold (i.e., more pain) when compared with baseline and sham after each injury and sustained up to 2 months (*n* = 10/group; ***p < 0.001, repeated-measures ANOVA, Tukey’s HSD). **C**, **D** Mouse grimace score (MGS) was assessed at baseline, 24 h after each injury, 3 days and 1 week in either sham animals (**C**) or injured animal group (**D**). No significant change in MGS was observed in the sham group; conversely, statistically significant increase in MGS (i.e., more pain) was determined at 24 h after each injury when compared with baseline. Increased MGS was sustained up to at least 1 week after the last injury (*n* = 7/group; *p < 0.05 **p < 0.01, ***p < 0.001, repeated-measures ANOVA, Tukey’s HSD)

To better understand the degree to which repetitive mTBIs affect the onset of allodynia, we subjected both male and female mice to von Frey mechanical stimuli. We observed a significant decrease in the tactile-dependent paw withdrawal threshold at 1 day after each injury compared to sham (Fig. 4). These mechanically evoked responses were evident in both the hindpaw ipsilateral (left) and contralateral (right) to the injured brain tissue (repeated-measure ANOVA, Fig. 4A, B). Mice were then assayed at multiple time points following cessation of the injury paradigm. Decreases in paw withdrawal threshold were sustained for at least two months in left hindpaw (Fig. 4A) and right hindpaw (Fig. 4B).

### Repetitive mild traumatic brain injury-induced changes in grimace scale as a measure of pain severity

MGS was used to assess spontaneous pain in sham and injured animals (Fig. 4C, D), where higher score indicates greater pain severity. The MGS assessment was performed at baseline, 24 h after each injury, 3 days and 1 week in both sham animals and injured animal groups. No significant change in MGS was observed in sham group (Fig. 4C); conversely, the injury group showed statistically significant increase in MGS (i.e., more pain) at 24 h after each injury when compared with baseline. The pain severity as measured by MGS was sustained for at least 1 week after the last injury.

### MCC950 dose dependently mitigates nociceptive sensitization and decreases activated (p20 fragment) caspase-1 protein at 24 h post-injury paradigm

NLRP3 antagonists have produced significant reductions in pain-like behaviors and associated inflammatory response [40–44]. Therein we investigated the degree to which a potent NLRP3-specific inhibitor known to penetrate the blood brain barrier [45], MCC950, altered nociceptive sensitization and caspase-1-mediated inflammatory response.

**Fig. 5:**
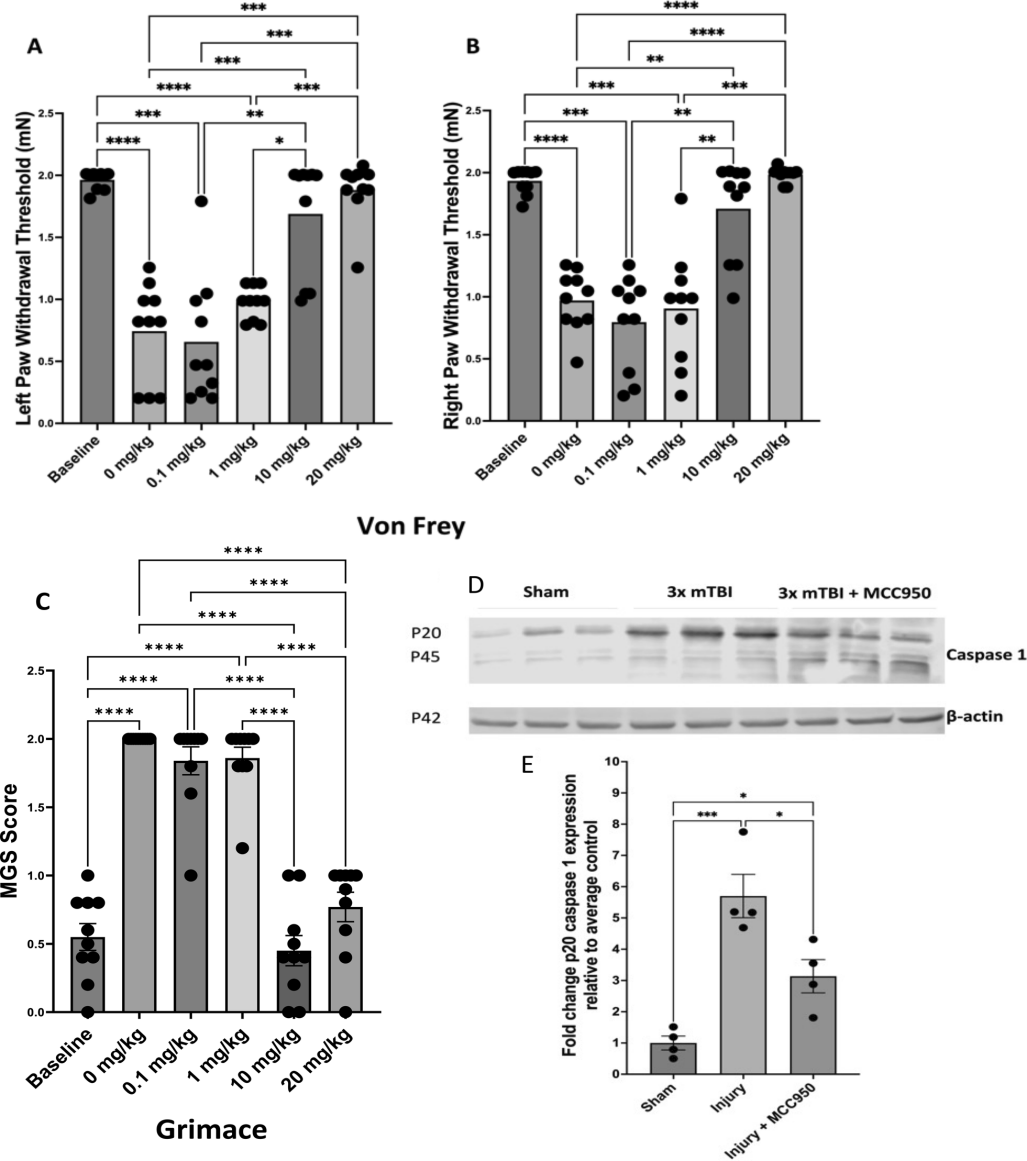
MCC950 dose dependently mitigates nociceptive sensitization and decreases activated (p20 fragment) caspase-1 protein at 24 h post-injury paradigm Hypersensitive hind paw withdrawal to tactile stimulus, ipsilateral to injured hemisphere (**A**, left hindpaw) and contralateral to injured hemisphere (**B**, right hindpaw), in injured animals at baseline, 24 h after three injuries, and 24 h after each injection dosage of MCC950. Statistically significant decrease in response thresholds (i.e., more pain) were observed at 24 h after three injuries when compared with baseline. There was no significant change in response threshold with 0.1 and 1 mg/kg treatment of MCC950 compared to before treatment. Conversely, significant increase was observed in response threshold (i.e., less pain) with 10 and 20 mg/kg MCC950 treatments (*n* = 10/group; *p < 0.05, **p < 0.01, ***p < 0.001, ****p < 0.0001, repeated-measures ANOVA, Tukey’s HSD) with no significant improvement at 20 mg/kg compared to 10 mg/kg. Mouse grimace scoring (MGS) was performed in animals at baseline, 24 h after last injury, and 24 h after each MCC950 injection dose. **C** Statistically significant increase in MGS (i.e., more pain) at 24 h after three injuries when compared with baseline in animals (*n* = 10/group; *p < 0.05, **p < 0.01, ***p < 0.001, ****p < 0.0001, repeated-measures ANOVA, Tukey’s HSD). No significant change in MGS scores with 0.1 and 1 mg/kg treatment of MCC950 compared to before treatment. Significant decrease in MGS scores (i.e., less pain) with 10 and 20 mg/kg MCC950 treatments (*p < 0.05, **p < 0.01, ***p < 0.001, ****p < 0.0001, repeated-measures ANOVA, Tukey’s HSD). No significant improvement at 20 mg/kg compared to 10 mg/kg. Western blot analyses of cortical caspase-1 levels revealed increased relative p20 caspase-1 expression after mTBI, which was decreased in the MCC950 treatment group (**D** and **E**). Mice were injected with MCC950 intraperitoneally at 10 mg/kg (5 mg/mL) at 1 h and 24 h after each injury. Cortical brain tissues were collected 24 h after the third injury. **D** Shown is a sample image of p20 caspase-1 activated protein levels in the sham, 3 × mTBI group, and 3 × mTBI group treated with MCC950. **E** Significant increases were detected in cortical activated p20 caspase-1 levels at 24 h after third mTBI in the injury group compared to sham. Injury animals treated with MCC950 showed significant attenuation of caspase-1 level (*n* = 4–5 mice per group; *p < 0.05, ***p < 0.001, two-way ANOVA, Tukey’s HSD)

Optimization of MCC950 dosing in vivo was determined using changes in both left (Fig. 5A) and right hindpaw withdrawal thresholds (Fig. 5B) and increases in grimace score 24 h after last mTBI injury using doses of 0.1, 1, 10, and 20 mg/kg. Optimum dosing was apparent in both males and females at 10 mg/kg MCC950 for allodynia and decreased grimace scores. A dose of 20 mg/kg did not produce a significant difference when compared to 10 mg/kg reagent dosing. These results suggest that 10 mg/kg of MCC950 adequately attenuated mTBI-induced nociceptive sensitization.

Immunoblotting of cortical brain tissue derived from mice subjected to the injury paradigm at 24 h revealed reduced levels of the activated (p20 fragment) caspase-1 protein in the MCC950 group (10 mg/kg) when compared to the increased p20 fragment levels in the vehicle-treated injury group (Fig. 5D, E).

### In vivo treatment with MCC950 resulted in reduction of caspase-1-mediated IVIS signals after repeated mTBI events

**Fig. 6:**
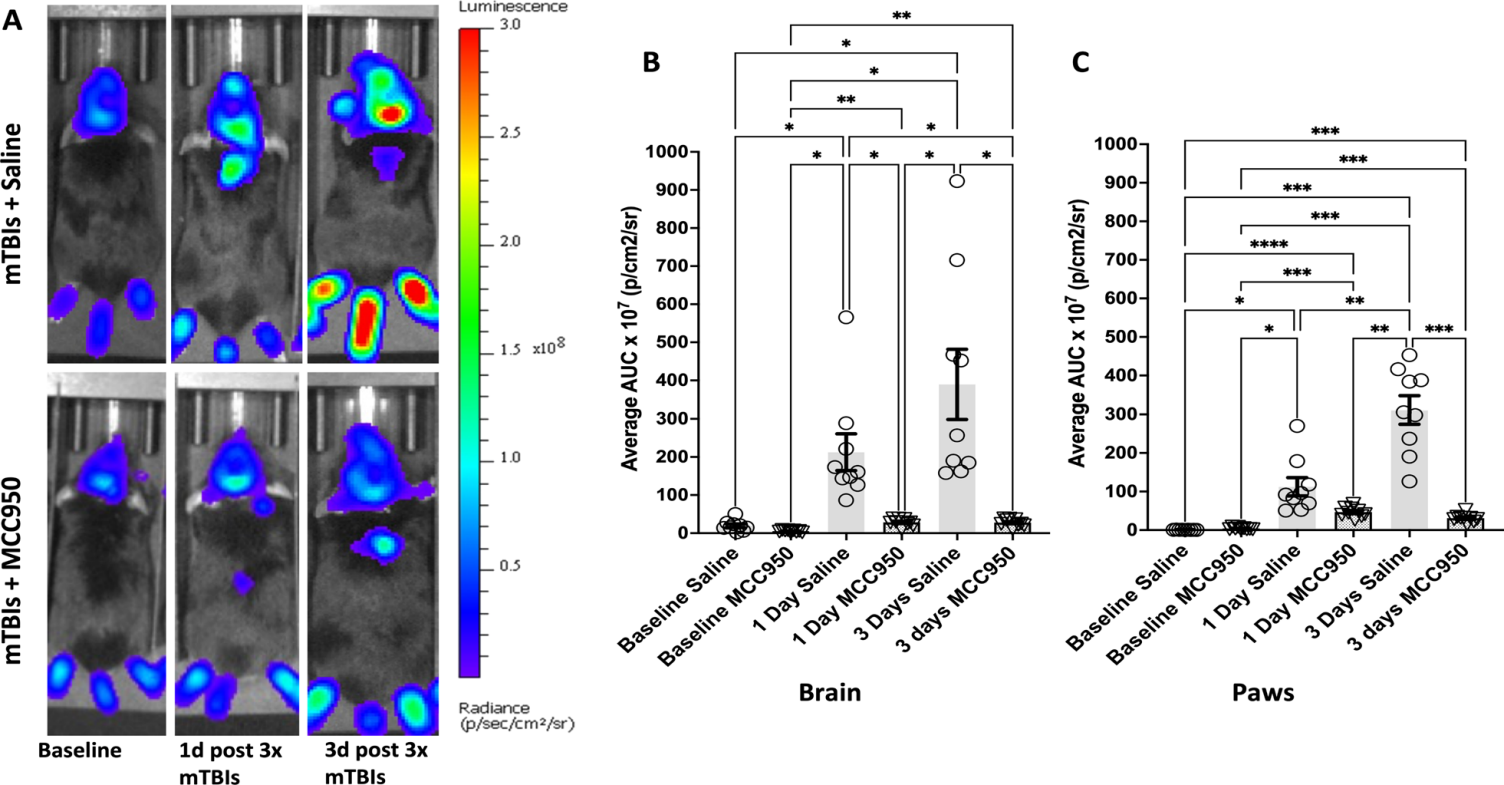
The NLRP3 inhibitor MCC950 blocked mTBI-induced caspase-1-mediated inflammatory signals in the brain and hindpaws of mice in vivo. **A** Sample images show caspase-1 activation reporter bioluminescence signals in mouse brains and hindpaws at 1 and 3 days after the third injury. Mice were either injected with saline (vehicle) or MCC950 intraperitoneally at 10 mg/kg (5 mg/mL) at 1 h and 24 h after each injury. The analyzed ROIs were quantified across all time points. **B–E** A significant decrease in caspase-1 activity signals was detected in the brain (**B**, **C**) and paw (**D**, **E**) of MCC950-treated animals (1 and 3 days) compared to the vehicle-treated group (*n* = 10–15 mice per group; *p < 0.05, **p < 0.01, ***p < 0.001, ****p < 0.0001, repeated-measure ANOVA, Tukey’s HSD)

We next determined whether the interruption of NLRP3 activation would influence mTBI-induced long-term inflammatory states. In these experiments, we treated mice with MCC950 intraperitoneally at 1 h and 24 h after each injury (total 6 treatments across injury timeline). Using IVIS imaging, we observed a significant reduction in bioluminescent signals at 1 day and 3 days after the repetitive mTBI events in both brain tissue and hind paws of MCC950 treatment groups compared to saline-treated groups (Fig. 6A–C).

**Fig. 7:**
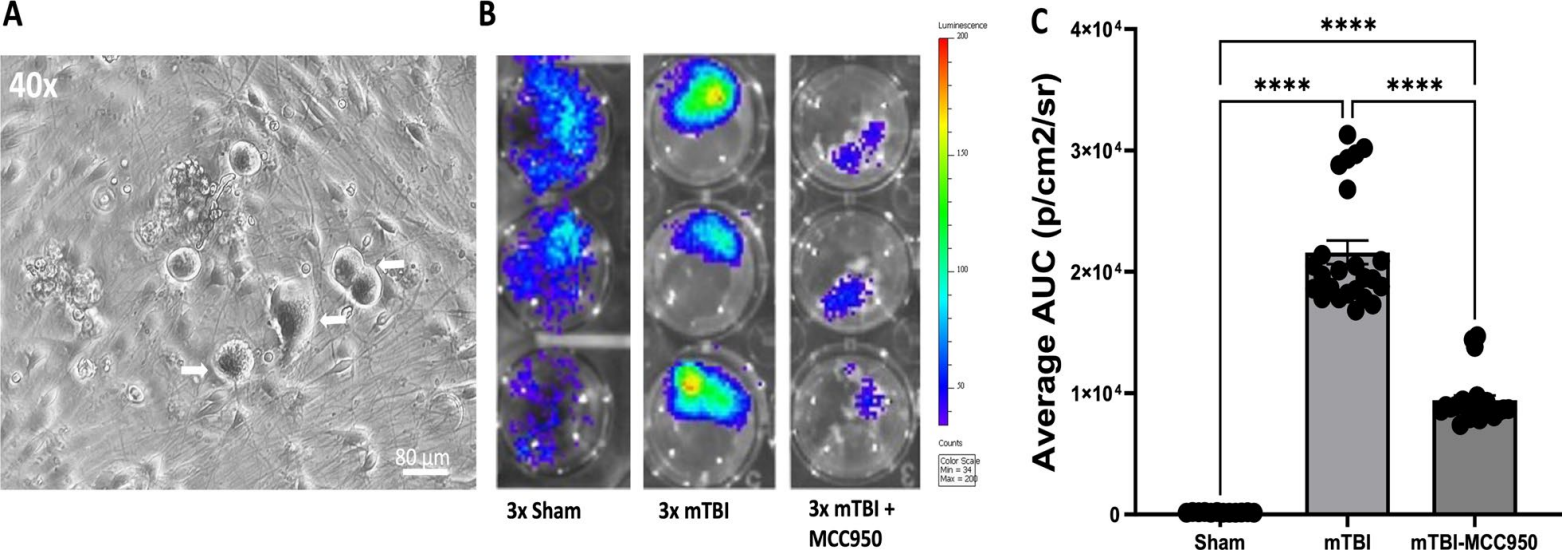
MCC950 reduced caspase-1-mediated inflammatory IVIS signal in in vitro cultured dorsal root ganglia (DRGs) of mTBI mice. Caspase-1 activation reporter mice were separated into three groups: sham injuries, 3 × mTBI injuries, and 3 × mTBI with MCC950 treatment. Animals underwent a series of three injuries (either sham or mTBI, each separated by one week) over the period of 3 week. Animals were treated with either saline or MCC950 after each injury. DRGs were then harvested at 24 h after the last injury and cultured. IVIS imaging was performed at three days after DRGs culture was established. **A** Sample image of cultured DRG at 3 days after plating showing primary sensory neurons (white arrows). **B** Sample images of IVIS imaging signals from cultured DRGs from sham, mTBI, and mTBI + MCC950 animals **C** Significant increase in caspase-1-mediated inflammatory signals in DRGs of mTBI animals compare to sham animals. This increase in inflammatory response was attenuated in injured animal that received MCC950 treatments (*n* = 4–5 animals per group; ~ 4 wells of 24-well plate per animal; ****p < 0.001, One-way ANOVA, Tukey’s HSD)

Moreover, IVIS imaging of in vitro cultured DRG cells derived from 3 × mTBI animals demonstrated a significantly increased in caspase-1 activation-associated bioluminescence signal compared to cultured DRG cells derived from 3 × sham animals (Fig. 7A–C). These signals, however, were significantly reduced in cultured DRG cells from 3 × mTBI animals exposed to MCC950 treatments (Fig. 7B, C).

### MCC950 treatment produced long-term changes in repetitive mTBI-associated mechanical allodynia and thermal hyperalgesia

**Fig. 8:**
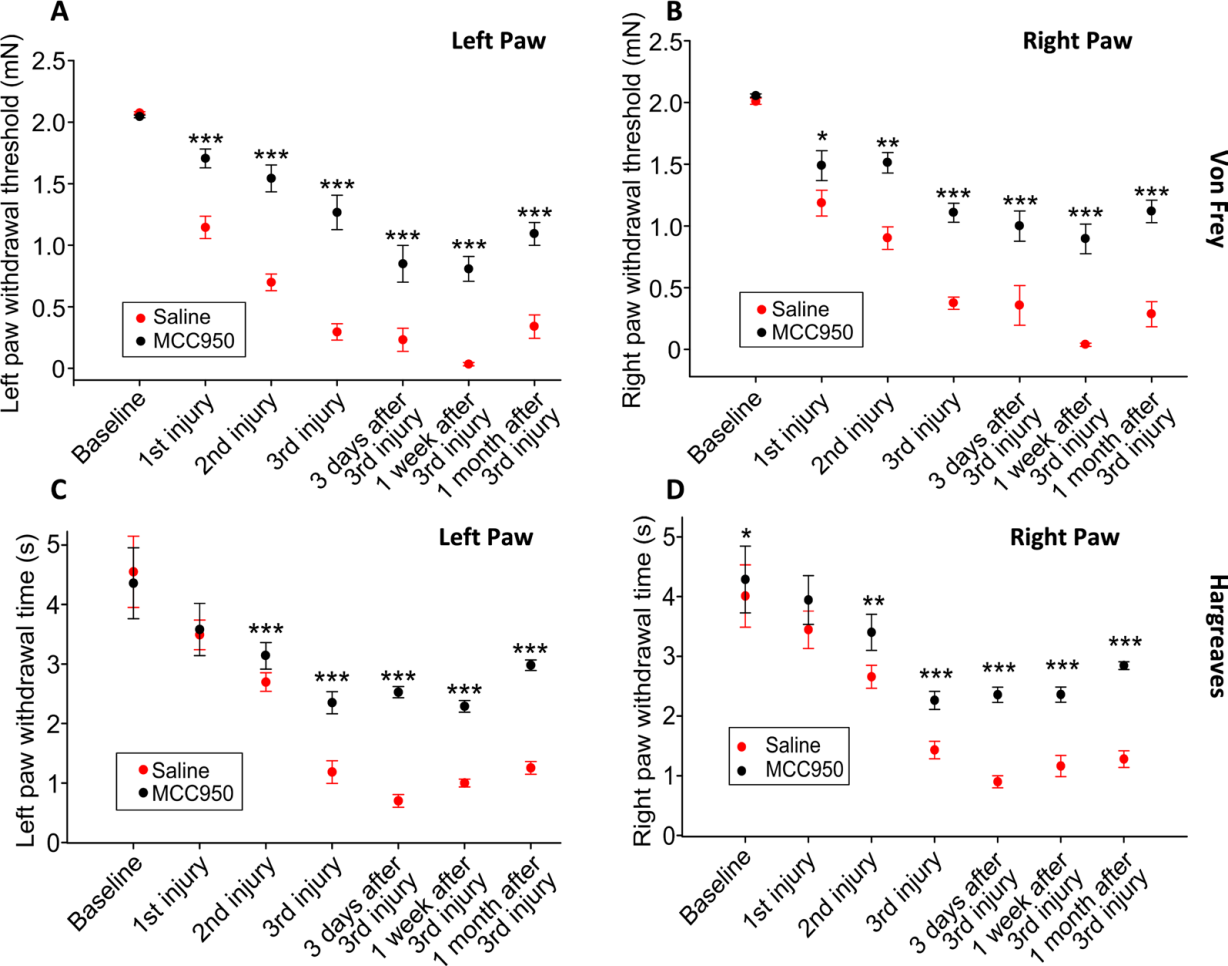
MCC950 treatments attenuated repetitive mTBI-induced long-term mechanical and thermal allodynia. Mice were injected with MCC950 intraperitoneally at 10 mg/kg (5 mg/mL) at 1 h and 24 h after each injury. There was greater hypersensitivity of hind paw withdrawal to tactile stimulus, ipsilateral to injury (left paw, **A**) and contralateral to injury (right paw, **B**), at 24 h after each injury and 3 days, 1 week, and 1 month after the third injury in saline-treated groups compared to MCC950-treated groups. A statistically significant increase in response thresholds (i.e., less pain) was detected in the MCC950-treated group after the second injury and was sustained up to 1 month after the third injury compared to the saline-treated group (*n* = 10–15 mice per group; *p < 0.05, **p < 0.01, ***p < 0.001, two-way ANOVA, Tukey’s HSD). Similar decrease in paw withdrawal time, bilaterally (**C** and **D**), of saline-treated injured animals compared to MCC950-treated injured animals were observed in Hargreaves thermal testing. Significant improvement of thermal pain response of MCC950-treated group was observed after 2^nd^ injury and sustain for one month compared to saline-treated group (*n* = 10–15 mice per group; *p < 0.05, **p < 0.01, ***p < 0.001, two-way ANOVA, Tukey’s HSD)

To explore whether MCC950 ameliorated changes in the nociceptive sensitization observed in mice subjected to repetitive mTBIs, we acutely treated mice after each injury with a 10 mg/kg i.p. injection of MCC950 or vehicle. Von Frey assays revealed increased bilateral hind paws response threshold to tactile stimulus at 24 h after each injury event (Fig. 8A, B). These behavioral reductions in pain sensitivity were bilaterally sustained up to one month (Fig. 8A, B). Similar observations were apparent with increased response time to noxious thermal stimulus (i.e., decrease pain sensitivity) in MCC950-treated injured animals compared to vehicle-treated mice (Fig. 8C, D).

## Discussion

This study used a closed-head injury mouse model of mTBI that mimics properties of human mTBI sequela. Despite its mild severity, changes in the acute inflammatory responses to repeated trauma can play an important role in the development and progression of a number of conditions, including nociceptive sensitization [46, 47]. In this study, we have demonstrated, using in vivo imaging, that repetitive closed-head mTBIs produce a significant increase in caspase-1-mediated inflammation, which is coincident with the development of long-term pain behavior states. By using the NLRP3 inflammasome inhibitor, MCC950, at early post-injury timepoints, we were able to decrease both injury-induced inflammatory responses as represented by attenuated caspase-1 activation and stimulus-dependent and independent behavioral changes in the mouse model.

The use of caspase-1 activation biosensor mice to monitor inflammatory responses in vivo validates the presence of brain inflammation following repetitive mTBI. This highly efficient technique provides a reproducible and effective approach to monitoring inflammatory response not only in vivo but also in ex vivo tissue preparations [20] and enables insights into the effects of these inflammatory responses on chronic behavioral pain states. These findings are consistent with previous animal studies which reported pronounced and robust increases in caspase-1 activation within the brain following head trauma [48]. In keeping with the prediction that inflammatory changes observed with repetitive mTBI contribute to chronic pain states, we also detected prolonged alterations in nociceptive sensitization for up to two months.

The simultaneous monitoring of changes in the brain tissue accompanying stimulus-dependent and independent pain behavior provides a more global view of the role that NLRP3 inflammasome plays in the development of persistent pain states. Although it is unknown to what degree the caspase-1-mediated signaling cascade and associated maturation of IL-1β or IL-18 contributes to the pathogenesis of pain states following mTBI [49], these proinflammatory cytokines have been identified in the TBI brain [16, 43, 50]. Indeed, IL-1β exerts its cellular effects by interacting with the cell surface (IL-1R) type I and type II receptors, with most effects mediated by IL-1RI, which in turn can elicit activation of p38 MAP [51]. Previous studies of pain models have also demonstrated that upregulation of glial cell IL-1β production, as a result of NLRP3-caspase-1 activation, affects several secondary pathways, such as inducing Cox-2 activation in neurons [52] and promoting phosphorylation of the NR1 subunit of NMDA receptors [53, 54].

The highly reproducible signaling activities of caspase-1 and downstream signaling are likely indicative of a tightly regimented inflammatory response to brain trauma. The degree to which IL-1b directly contributes to peripheral tissue nociceptive sensitization is generally unknown but may involve cyclooxygenase (COX) upregulation within peripheral sensory ganglia inducing neuronal sensitization [55]. The acute phases of mTBI-associated inflammation may effectively stimulate the generation and release of several proinflammatory cytokines into the systemic circulation which may serve to induce neuronal sensitization in other anatomical levels such as cells in the DRG [56]. Alternatively, chronic inflammation due to mTBI may serve to disrupt descending noradrenergic and serotonergic pain control circuits and contribute to the loss of diffuse antinociceptive inhibitory control [57–59].

Our goal was to use a caspase-1 activation biosensor to monitor inflammatory responses following repetitive mTBI in vivo. One caveat to using transgenic mice expressing global biosensors such as the firefly luciferase reporter gene is the lack of cell specificity responsible for the resulting bioluminescent signal. Therefore, the observed bioluminescence signal is only a measure of average promoter activity over the entire sampled cell population but is no measure of the variation in promoter activity that exists between individual cells in the population or the specificity of cell types which may ultimately be responsible for the associated injury-induced inflammatory or neuroinflammatory responses. There is a distinct possibility that some of the cell types responsible for the caspase-1 activation following repetitive mTBI could include bone marrow-derived macrophages [60, 61]. Given the long-term changes in innate immune cell populations following repetitive mTBI paradigm [60, 61], the transfer of peripheral blood mononuclear cells or bone marrow stem cells expressing the caspase-1 activation reporter into wild type recipient mice would facilitate monitoring of biosensor activation in specific immune cell populations.

## Conclusion

These observations herein strongly suggest that a caspase-1/NLRP3-dependent mechanism may underlie mTBI-induced long-term nociceptive sensitization. More importantly, the increased caspase-1 biosensor activation observed in our model system and the presumptive increase of mature IL-1β release perhaps can explain some of the beneficial behavioral impacts observed following treatments with the receptor IL-1R1 neutralizing antibody [62] and IL-1β [63] neutralizing antibody. The MCC950 treatment paradigm utilized in this study suggests that modulating upstream signaling of the inflammasome-caspase-1 activation pathway is critical to preventing the development of injury-associated changes in nociceptive sensitization. Taken together, the caspase-1 activation reporter mouse model allows real-time monitoring of the severity of repetitive mTBI-associated inflammation in the in vivo setting and can yield relatively precise spatial measurements of the inflammatory changes due to both injury and disease.

### Supplementary Information

**Additional file 1: Fig. S1.** Ex vivo IVIS imaging of caspase-1 activation in freshly cut brain slices from caspase-1 activation reporter mice subjected to either the Sham or mTBI procedure. Brains from sham control, 1-day post-injury, and 3 days post-injury mice were sectioned into ~ 400 µm thick slices and incubated in the standard ACSF solution as described in the Methods. Images were obtained 1 h after preparation and one minutes after addition of 100 µL D-luciferin (at 20 mg/ml) to the bath solution. A. Sample images of brain slices and IVIS bioluminescence responses of control and different time groups. B. Significant increases in bioluminescence signals were observed in brain slices of 1 day and 3 days mTBI animals as compared to controls. *n* = 3–4 animals per group; ~ 15–20 slices per animal), **p < 0.01, ***p < 0.005 One-way ANOVA, Tukey’s HSD.

## Data Availability

All data and materials are available from the corresponding author upon request.

## Abbreviations

mTBI: Mild traumatic brain injury
DAMPs: Damage-associated molecular patterns
NLR: NOD-like receptors
NLRP3: Nucleotide-binding oligomerization domain (NOD)-like receptor 3
CCI: Control cortical impact
IOP: Intraocular pressure
BLI: Bioluminescence imaging
IVIS: In Vivo Imaging System
IBA1: Ionized calcium binding adaptor protein 1
GFAP: Glial fibrillary acidic protein
DAPI: 4’,6-Diamidino-2-phenylindole
